# Associations Between Health-Related Quality of Life and Clinical Factors in Lumbar Disc Herniation: Evidence from a Romanian Cohort Using the SF-36

**DOI:** 10.3390/jcm14228258

**Published:** 2025-11-20

**Authors:** Mădălina Duceac (Covrig), Salim Camer, Irina Luciana Gurzu, Alina Pleșea-Condratovici, Liviu Stafie, Letiția Doina Duceac, Lucian Eva, Bogdan Gurzu, Mădălina Nicoleta Matei, Ciprian Adrian Dinu, Cristian Guțu, Doina Carina Voinescu

**Affiliations:** 1 Doctoral School of Biomedical Sciences, “Dunărea de Jos” University of Galați, 47 Domnească Street, 800008 Galați, Romania; madalinaduceac@yahoo.ro; 2 “Prof. Dr. Nicolae Oblu” Emergency Clinical Hospital, 2 Ateneului Street, 700309 Iași, Romania; letimedr@yahoo.com (L.D.D.); elucian73@yahoo.com (L.E.); 3 Faculty of Medicine, Ovidius University of Constanta, 1 University Street, 900470 Constanța, Romania; salimcamer@yahoo.com; 4 Department of Preventive Medicine and Interdisciplinarity, Faculty of Medicine, Grigore T. Popa University of Medicine and Pharmacy, 700115 Iași, Romania; liviu.stafie@umfiasi.ro; 5 Faculty of Medicine and Pharmacy, “Dunărea de Jos” University of Galați, 47 Domnească Street, 800008 Galați, Romania; madalina.matei@ugal.ro (M.N.M.); ciprian.dinu@ugal.ro (C.A.D.); cristian.gutu@ugal.ro (C.G.); carinavoinescu@gmail.com (D.C.V.); 6 Department of Morfofunctional Sciences II, Faculty of Medicine, Grigore T. Popa University of Medicine and Pharmacy, 700115 Iași, Romania; bgurzu@yahoo.com

**Keywords:** lumbar disc herniation, SF-36, health-related quality of life, activity limitation, functional capacity, risk factors, pain, population study

## Abstract

**Background**: Lumbar disc herniation (LDH) has a substantial impact on health-related quality of life (HRQoL), mainly through pain, reduced mobility, and functional limitations. To better reflect these outcomes, we utilized the SF-36 survey, a well-established tool commonly used in both clinical and population research. The purpose was twofold: to evaluate HRQoL and to explore the main factors linked with activity limitation in a community-based group of Romanian patients. **Methods**: This cross-sectional study included 120 participants with LDH who completed the SF-36 questionnaire. Internal consistency was high (Cronbach’s α = 0.922). Descriptive statistics, Chi-square tests, *t*-tests, and multivariate regressions were performed using SPSS 18.0. **Results**: Of the respondents, 53.3% were female and 58.4% were over 60 years old. While 55% reported good or very good health, 26.7% had severe Daily Activity Limitation (DAL) based on the SF-36 physical function score, and 62.5% scored in the poorer range (33–45) of the Physical and Emotional Status (PES) composite derived from the SF-36. Female sex and age over 60 were each associated with nearly a twofold-higher risk of moderate-to-severe DAL (OR = 2.20, 95% CI: 1.04–4.65), while reporting severe or very severe pain increased this risk more than fourfold (OR = 4.64, 95% CI: 1.89–24.21). **Conclusions**: In patients with LDH, poor self-rated health, older age, female sex, and high pain intensity were significantly associated with reduced functional capacity. The SF-36 proved to be a valuable tool for identifying vulnerable subgroups that require targeted rehabilitation and psychosocial support. From a public health perspective, these findings also support the use of SF-36 in community screening and in guiding resource allocation for multidisciplinary rehabilitation programs.

## 1. Introduction

The 36-Item Short Form Health Survey (SF-36) is one of the most frequently used instruments for measuring health-related quality of life (HRQoL) in both clinical practice and population research [1,2]. Published data confirmed its reliability, validity, and cross-cultural adaptability [3,4,5,6].

The SF-36 is a validated and adaptable instrument for assessing health-related quality of life, demonstrating consistent psychometric performance across clinical conditions—including musculoskeletal, renal, and endocrine disorders—and has been validated and applied in Romanian populations across a range of clinical contexts [7,8,9,10].

Despite this body of work, the SF-36v2 has not yet been tested in Romanian patients with LDH, nor have studies examined its relationship with demographic and clinical characteristics such as age, gender, or pain severity. The present study addresses this gap by evaluating the internal reliability and feasibility of the SF-36v2 in LDH patients, reporting new data on self-perceived HRQoL, and identifying demographic and clinical factors associated with functional limitations. To our knowledge, this is the first systematic assessment of HRQoL in Romanian LDH patients using the SF-36.

## 2. Materials and Methods

This cross-sectional observational study aimed to evaluate health-related quality of life (HRQoL) in Romanian adults diagnosed with LDH. A total of 120 patients were enrolled and voluntarily completed the SF-36 Health Survey using a secure online platform. Recruitment was conducted through clinical networks and patient support groups, and all responses were collected anonymously. All participants were diagnosed with lumbar disc herniation and met the inclusion criteria (age over 18, confirmed diagnosis). The study protocol was approved by the Ethics Committee of Clinical Hospital of Emergency “Prof. Dr. Nicolae Oblu” Iasi (approval no. 2/23 February 2023), and all participants provided informed consent. The minimum sample size was determined using an online calculator (INFOmass SRL, Iași, Romania) based on a target population of 988,981 individuals, a confidence level of 95%, and a 9% margin of error, which indicated that 120 participants would be sufficient. This calculation was used to guide recruitment feasibility; however, the resulting cohort represents a community-based convenience sample of patients with confirmed LDH rather than a random population-based sample. This recruitment strategy may introduce a degree of selection bias; however, it also ensured inclusion of patients from diverse community backgrounds outside of a single hospital setting, thus enhancing the ecological validity and real-world relevance of the findings.

The SF-36 is a multidimensional instrument that assesses eight domains of perceived health: physical functioning, role limitations due to physical health, bodily pain, general health perceptions, vitality, social functioning, role limitations due to emotional problems, and mental health. Individual domain scores were computed from raw values and then standardized. In this scoring system, lower values indicate greater functional limitation, whereas higher scores reflect better perceived health status.

All participants received the same version of the SF-36, and responses to items concerning limitations in daily activities were scored as 1 (very much), 2 (somewhat), or 3 (not at all). These raw scores were then standardized, with lower values denoting greater perceived limitation [10]. This refers to the raw scoring of specific items prior to transformation into standardized SF-36 domain scores. In addition to the standard SF-36 domains, we derived a DAL score by summing the 10 physical functioning items (each scored 1–3). The DAL scale ranged from 10 to 30, with lower scores indicating greater limitation, and was categorized as severe (10–14), moderate (15–23), or mild (24–30). We also created a composite PES score, based on selected items from the vitality and mental health domains, to capture overall psychosocial well-being. The PES score ranged from 22 to 45, with higher scores reflecting poorer psychosocial well-being. Although not a standard SF-36 output, this composite was developed as an exploratory indicator of combined physical vitality and emotional status. Unlike the validated Physical and Mental Component Summary scores (PCS and MCS), which are derived using weighted algorithms from all SF-36 domains [1,2], the PES score was created ad hoc and is not part of the standard scoring framework. No psychometric validation was attempted in this study; the PES score was used solely for exploratory purposes.

Categorical variables were examined using non-parametric tests, including the Chi-square and Kruskal–Wallis tests, to compare frequency distributions across subgroups. Normality of quantitative variables was assessed using the Kolmogorov–Smirnov test, which is suitable for larger samples. A threshold of *p* > 0.05 was used to retain the null hypothesis, suggesting that the data were normally distributed. Additional indicators such as kurtosis were also analyzed to evaluate distributional normality and homogeneity.

Comparisons between group means were conducted using Student’s *t*-test for normally distributed data. For non-parametric comparisons, Kruskal–Wallis tests were applied for more than two groups, while Mann–Whitney U tests were used for two-group comparisons when normality assumptions were not met.

To assess the relationship between HRQoL outcomes and potential predictors, multivariate analyses were performed. Linear regression using the least squares method was applied to identify associations between a single dependent variable and multiple independent variables. Independent variables included age, sex, and pain severity, while HRQoL domain scores were treated as dependent variables. For logistic regression, we defined the outcome as moderate-to-severe daily activity limitation (DAL score ≤ 14 coded as 1 vs. mild limitation coded as 0). Age, sex, and pain severity were entered as independent variables. All predictors were included as main effects without interaction terms, given the limited sample size.

Regression assumptions were checked; residual plots for linear models showed no major deviations from linearity, normality, or homoscedasticity, and variance inflation factors (VIFs) for logistic regression were <2, indicating no concerning multicollinearity. Internal consistency of the SF-36 scale was verified using Cronbach’s alpha, which yielded a value of 0.922, well above the accepted reliability threshold of 0.70. All statistical procedures were performed using SPSS version 18.0 (IBM Corp., Armonk, NY, USA), with a significance level set at *p* < 0.05. Smaller *p*-values were interpreted as stronger evidence against the null hypothesis.

## 3. Results

Detailed stratified results by sex and age for all SF-36 domains (Q1–Q8) are available in Supplementary Table S1.

### 3.1. Q1-Self-Rated Health (SRH)

Respondents rated their general health on a five-point scale. Most described their health as *good* (35%) or *very good* (20%), while 27.5% reported *fair* and 10.8% *poor health*. Only 6.7% rated their health as excellent. Sex- and age-specific analyses (Supplementary Table S1) revealed that poor SRH was disproportionately reported by women (61.5%, *p* = 0.028) and by those older than 60 years (84.6%, *p* = 0.036). Conversely, men predominated in the *excellent* and *very good* categories, and younger adults more often reported better health.

### 3.2. Q2-Health Compared with One Year Ago

When asked to compare their health with the previous year, 28.3% reported being *much better* and 20.0% *somewhat better*, while 24.2% indicated *no change*. By contrast, 18.3% felt *somewhat worse* and 9.2% *much worse*. Demographic patterns were pronounced (Supplementary Table S1): improvements were most often reported by men (67.6%, *p* = 0.001) and younger adults (50% ≤ 60 years, *p* = 0.011), while declines were predominantly reported by women (90.9%, *p* = 0.001) and participants over 60 years (81.8%, *p* = 0.011).

### 3.3. Q3-Daily Activity Limitation

**Figure 1:**
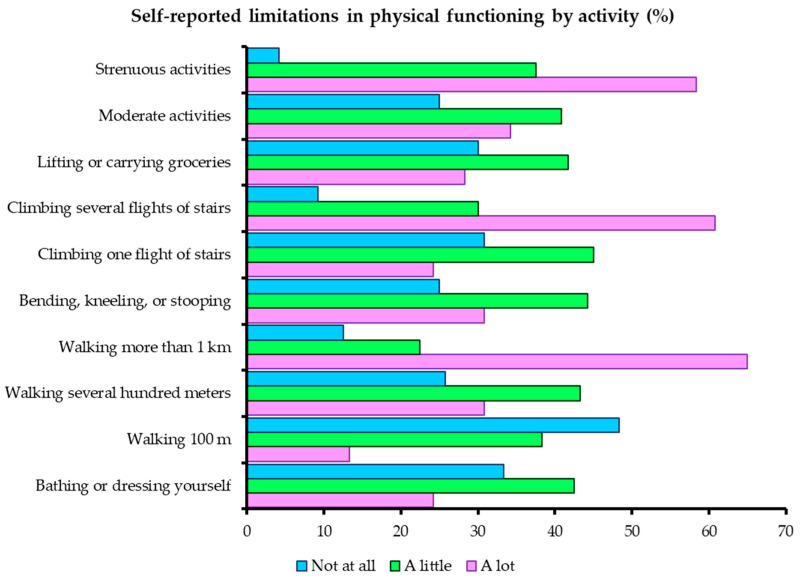
Self-reported limitations in physical functioning by activity (%).

Limitations were most frequently reported for walking more than 1 km (65%), climbing several flights of stairs (60.8%), and performing strenuous activities (58.3%), while bathing, dressing, and short-distance walking were largely preserved (Figure 1).

The DAL score ranged from 10 to 30 (mean = 18.74, SD = 5.67; median = 17) and followed an approximately normal distribution (Kurtosis = 1.79; *p* > 0.05, Kolmogorov–Smirnov test), supporting the use of parametric analysis. Participants were classified into mild, moderate, and severe limitation subgroups, comprising 25.0%, 48.3%, and 26.7% of the cohort, respectively.

A total of 32 patients (26.7%) had a limitation score between 10 and 14, indicating severe limitation; 58 patients scored between 15 and 23, corresponding to moderate limitation; and 30 patients had scores between 24 and 30, reflecting mild limitation (Table 1). Sex and age distributions showed that severe DAL was more common among women (56.3%) and older adults (56.3%, *p* = 0.031). In this group, 40.6% reported fair SRH and 37.5% indicated worsening health compared to the previous year (Table 1). Conversely, mild DAL was more frequent in younger participants, 56.7% of whom rated their SRH as good.

Regression analysis confirmed that age, SRH, and perceived health change together explained 16.6% of DAL variance (Adjusted R^2^ = 0.166, *p* = 0.001; Table 2).

The final model estimated DAL as (Table 3):

Y = 2.98 − 0.001 × Age − 0.224 × SRH − 0.086 × Health Change

Mean SF-36 Physical Function score was 45 in those >60 vs. 55 in ≤60 (*p* < 0.01), a 10-point difference on the 0–100 scale, suggesting substantial functional decline with age.

### 3.4. Q4-Role Limitations Due to Physical Health

Most participants reported restrictions in their daily or occupational roles during the past four weeks: 76.7% performed fewer activities, 74.2% had difficulties completing tasks, 73.3% reduced working time, and 65.8% faced limitations in the type of work performed. While sex- and age-related differences were modest, associations with DAL severity were highly significant (Supplementary Table S1). All patients with severe DAL reduced their working time and reported fewer activities and difficulties at work; 93.8% also experienced limitations in the type of work they could perform.

Among those with moderate DAL, 70–83% reported similar restrictions, compared with 30–43% in the mild DAL group (Table 4).

### 3.5. Q5-Role Limitations Due to Emotional Problems

Emotional health also interfered with role functioning. About 73% of respondents reported reduced working time, fewer activities, or difficulties maintaining attention due to emotional problems. Although sex- and age-related differences were not statistically significant, full results are available in Supplementary Table S1.

However, DAL severity showed strong correlations: over 90% of patients with severe DAL experienced limitations across all role limitations due to emotional problems items, compared to ~80% in the moderate group and 40% in the mild group (Table 5).

### 3.6. Q6-Social Functioning

Health-related problems substantially impaired social life. Nearly half of participants reported that their social activities with family, friends, or neighbors were affected a lot (30%) or very much (20%), while only 7.5% reported no impact. Although sex differences were minimal, DAL severity strongly predicted social functioning outcomes (Supplementary Table S1).

In the severe DAL group, 43.8% reported that social activities were affected very much and 34.4% a lot. In contrast, 43.3% of those with mild DAL reported only slight impairment, and 23.3% no impact (Table 6).

### 3.7. Q7-Bodily Pain

Pain intensity was high in this cohort. One-third of participants reported severe pain, 32.5% moderate, and 13.3% very severe, while only 2.5% reported no pain. Among those reporting severe pain, 57.5% were women, whereas among those reporting very severe pain, 62.5% were men and 56.3% were younger than 60 years (see Supplementary Table S1).

DAL severity correlated closely with pain: 56.3% of those with severe DAL reported severe pain, and 28.1% very severe pain. By contrast, patients with mild DAL most often reported very mild or moderate pain (Table 7).

### 3.8. Q8-Pain Interference with Work

Bodily pain significantly affected work and daily activities. A total of 36.7% of participants reported that pain interfered with usual work a lot, and 15.8% very much, while only 5% reported no effect. Demographic differences were not significant; full results are provided in Supplementary Table S1.

However, DAL severity showed strong associations: 87.5% of those with severe DAL reported that pain interfered with work a lot or very much, compared to 79.3% in the moderate group and only 6.7% in the mild group (Table 8).

Logistic regression confirmed that age > 60 doubled the risk of severe or moderate DAL (OR = 2.20, 95% CI: 1.04–4.65), while reporting severe or very severe (‘intense’) pain increased this risk more than fourfold (OR = 4.64, 95% CI: 1.89–24.21). Female sex was also entered into the model but was not statistically significant.

### 3.9. Q9-Vitality and Emotional Well-Being

**Figure 2:**
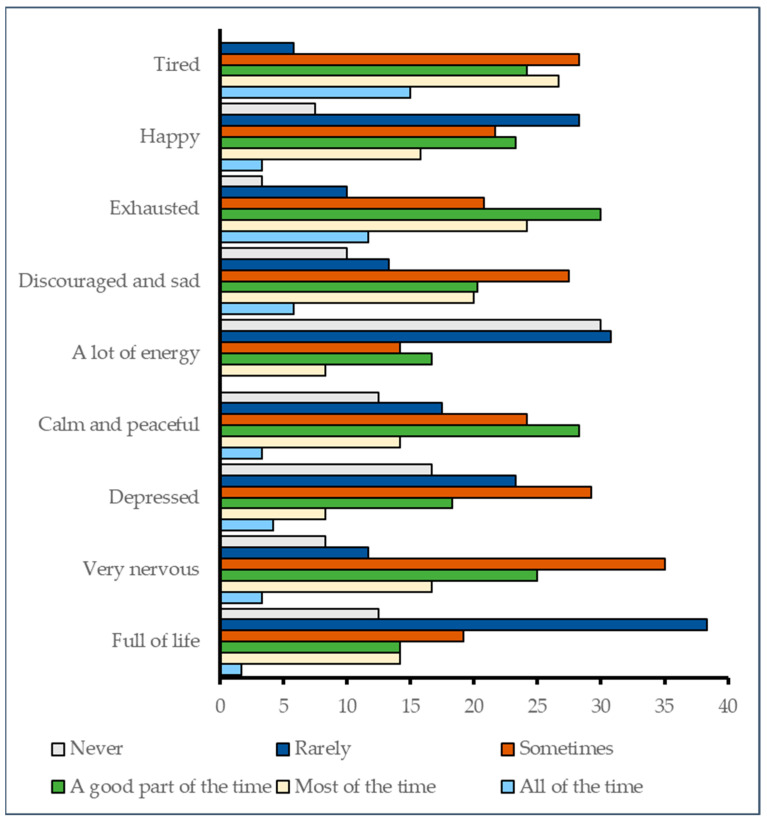
Self-reported physical and emotional status in the past four weeks (%).

Participants frequently reported reduced vitality and psychological strain. As shown in Figure 2, 38.3% rarely felt full of life, 30.8% rarely had energy, and 30% stated they never had energy. Negative emotions were common: 35% felt very nervous, 29.2% sometimes depressed, and 27.5% discouraged or sad.

The composite PES score ranged from 22 to 45 (mean = 33.36, SD = 3.27), with over 60% of participants (62.5%) scoring in the poorer range (33–45), indicating reduced vitality and emotional well-being. The distribution approximated normality (*p* > 0.05, Kolmogorov–Smirnov test; Kurtosis = 1.839), supporting the use of parametric analysis.

As noted in the Methods, the PES score was derived as an exploratory composite from SF-36 vitality and mental health domains to provide a summary indicator of psychophysical well-being. It served to characterize how physical vitality and emotional functioning manifested together in this patient group. While the PES demonstrated internal consistency in terms of expected associations with pain and social participation, it has not undergone psychometric validation and should be interpreted cautiously as an exploratory construct. While demographic associations were nonsignificant, poorer PES scores were significantly associated with worse pain, reduced social functioning, and lower likelihood of perceived health improvement (Table 9).

Regression analyses showed that conventional predictors explained very little variance in PES (Adjusted R^2^ = 0.008, *p* = 0.143), suggesting that unmeasured psychosocial factors may be more influential (Table 10).

### 3.10. Q10-Interference of Health with Social Activities

When asked how physical or emotional health interfered with social activities such as visiting friends or relatives, 5.0% of participants reported being affected all of the time, 15.0% most of the time, and 25.0% sometimes. An additional 32.5% reported being rarely affected, and 22.5% not at all. Overall, nearly half of the respondents (45%) experienced at least some degree of social restriction.

Compared with other SF-36 domains, social functioning appeared somewhat less impaired, as more than half of respondents were rarely or never affected. This may reflect coping strategies or prioritization of social roles despite health limitations.

### 3.11. Q11-General Health (GH) Perceptions

**Figure 3:**
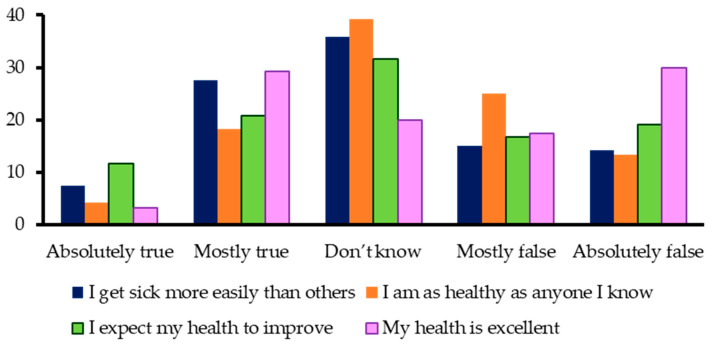
Answers to the question regarding health status.

The GH domain reflected widespread pessimism. In response to the statement “My health is excellent”, 30% of participants answered absolutely false and 17.5% mostly false, meaning nearly half of the sample perceived their health as poor (Figure 3).

Detailed subgroup analysis confirmed that poorer GH perceptions were significantly associated with female sex (*p* = 0.031), age > 60 years (*p* = 0.001), fair or poor SRH (*p* = 0.001), perceived decline in health compared with one year ago (*p* = 0.01), DAL severity (*p* = 0.001), and substantial social impairments (*p* = 0.001) (Table 11, Table 12 and Table 13).

Additional GH items revealed that one-third of participants expected their health to deteriorate, and a similar proportion perceived themselves as less healthy than peers or more susceptible to illness (Figure 3). These negative expectations were again closely linked with older age, female sex, higher DAL, and impaired social functioning (Table 12, Table 13 and Table 14).

The following Discussion interprets these findings in light of prior literature and clinical practice, with particular attention to pain, daily role functioning, and social participation.

## 4. Discussion

This study assessed health-related quality of life (HRQoL) in patients with LDH using the SF-36 instrument. The findings reveal a multidimensional burden affecting physical, emotional, and social functioning, with strong associations between pain, demographic characteristics, and perceived health.

### 4.1. Self-Rated Health and Health Change

Most respondents rated their health as good or very good, although more than one-third described it as fair or poor (see Supplementary Table S1). Women and older adults were disproportionately represented in these categories (see Supplementary Table S1). Compared with European data showing fewer than 15% reporting poor health [11,12], our cohort appears more negatively skewed. Similarly, while nearly half of participants reported improved health compared to one year ago, 27.5% perceived deterioration (see Supplementary Table S1), with older women most affected (see Supplementary Table S1). These findings mirror international data confirming that women and older adults are more likely to report worsening health [13,14,15]. In our cohort, nearly 91% of those who reported health deterioration over the past year were older women, underscoring this subgroup as particularly vulnerable to HRQoL decline. Such findings suggest that targeted interventions for older women with LDH may be especially important.

### 4.2. Physical Functioning and Daily Activity Limitation

Severe restrictions were reported in walking >1 km, climbing stairs, and strenuous activities, while self-care remained preserved (Figure 1). DAL classification (mild/moderate/severe) showed a near-normal distribution and was used in subgroup analyses. DAL severity was strongly associated with age, SRH, and perceived health change (Table 1). This aligns with prior evidence linking age, comorbidity, and depressive symptoms to declines in SF-36 physical functioning [13,16,17]. The regression analyses underscore the central role of pain in limiting daily activities among LDH patients. While age and self-rated health contributed to DAL variance, together they explained only 16.6% of the outcome, highlighting the modest explanatory power of the model and the likely influence of additional unmeasured factors such as comorbidities, psychosocial status, and occupational demands. Our results require cautious interpretation given the lack of multiple comparison adjustments; marginal associations in particular should be treated as hypothesis-generating. Future studies should systematically assess comorbidity profiles, socioeconomic status, and psychological factors to more comprehensively capture the determinants of HRQoL in patients with LDH. In the logistic model, both older age and reporting severe or very severe (“intense”) pain were independently associated with a higher likelihood of moderate-to-severe DAL, consistent with international findings. Female sex, although associated with lower HRQoL in univariate analyses, did not remain significant once age and pain were taken into account, suggesting that the observed sex effect may be mediated through these variables. To our knowledge, this is the first study in Romania to demonstrate these associations in an LDH population using the SF-36. Future studies should stratify patients by pain duration and chronicity to better understand how these factors influence HRQoL and rehabilitation needs.

### 4.3. Role Functioning (Physical and Emotional)

Role limitations due to physical health were widespread: most participants reported fewer activities, reduced working time, and difficulties at work (see Supplementary Table S1), with strong associations to DAL severity (Table 4). Similarly, role limitations due to emotional problems limitations were highly prevalent, with about 70% reporting reduced work time or impaired concentration due to emotional problems (see Supplementary Table S1). Normative data from Switzerland and Hungary indicate much higher role limitations due to physical health score and role limitations due to emotional problems scores in the general population [14,15]. Role functioning, social participation, and work performance were significantly more restricted in patients with more severe DAL. This pattern shows that difficulty with daily activities isn’t just about physical limitations, it affects emotional well-being and social connections too.

### 4.4. Social Functioning and Interference with Social Activities

Nearly half reported that social activities were affected a lot or very much (see Supplementary Table S1). When specifically asked about interference with visiting friends or relatives, 45% reported at least some restriction (Supplementary Table S1). Compared with normative data showing mean social functioning scores above 80 [13,14,15], these findings highlight the social burden of LDH.

### 4.5. Pain and Its Impact on Work

One-third of participants reported severe pain and 13% very severe pain (see Supplementary Table S1). Pain also interfered substantially with usual work, with 36.7% of participants reporting that it affected them a lot and 15.8% very much (see Supplementary Tables S1, 7 and 8). Logistic regression confirmed that age > 60 doubled the risk of severe DAL, while reporting severe or very severe (“intense”) pain increased this risk more than fourfold. These results are in line with earlier research that connected pain to lower HRQoL in a variety of neurological and musculoskeletal disorders [13,18].

An interesting nuance was that very severe pain was more frequently reported by men in our cohort, diverging from much of the musculoskeletal pain literature in which women typically report higher pain levels. One possible explanation may be cultural differences in pain reporting, with men endorsing “very severe” pain only when discomfort is extreme. Consistent with prior studies in chronic low back pain, we observed associations between higher pain intensity, female sex, and reduced SF-36 scores, findings that parallel those from chronic low back pain studies and demonstrate their applicability in Romanian patients [10,19,20,21]. Investigating psychosocial factors and gender-based reporting differences will help disentangle whether our findings reflect cultural pain expression norms or unmeasured gender-specific influences.

### 4.6. Vitality and Emotional Well-Being

As described, the PES score reflected reduced vitality and emotional well-being in over 60% of the cohort, with common reports of fatigue, nervousness, and discouragement. Lower PES was significantly associated with higher pain and reduced social participation (Table 9), but the model explained only a small fraction of its variability, pointing to missing psychosocial influences (Table 10). Although the PES score demonstrated internal consistency in terms of distributional characteristics and expected associations with HRQoL domains, it has not undergone formal psychometric validation (e.g., factor analysis or reliability testing) and should therefore be interpreted as an exploratory measure.

### 4.7. General Health Perceptions

Self-rated health and GH items revealed consistently pessimistic perceptions, especially among older women—mirroring trends reported under both SRH and DAL outcomes. Nearly half rejected the statement “My health is excellent” (Figure 3), with poorer GH perceptions associated with female sex, older age, DAL severity, and impaired social participation (Table 11, Table 12, Table 13 and Table 14). Compared with normative Swiss and Hungarian populations, GH scores were markedly worse [14,15]. Such negative expectations have been shown to predict morbidity and mortality [22].

### 4.8. Broader Context and Implications

These findings parallel chronic low back pain (CLBP) literature, where pain intensity, age, and female sex are consistently associated with lower SF-36 scores [23,24]. Domains most affected in our study, role-physical, bodily pain, and vitality, are also consistently impaired in CLBP, and interventions such as core stability or therapeutic climbing have demonstrated improvements [25,26]. While the SF-36 provided a broad assessment of health-related quality of life, it is a general instrument and may not capture the full extent of spine-specific disability. A lumbar-specific instrument like the Oswestry Disability Index would have strengthened our assessment by capturing functional limitations tied more directly to disc herniation. This dual approach, using both a generic and a condition-specific tool, has been shown to yield complementary information in spine patients [27]. We chose the SF-36 for its wide applicability and validation across populations; however, we acknowledge that a disease-specific scale like ODI might have highlighted subtle disabilities and improved the nuance of our results. Although our findings provide useful insight into HRQoL in Romanian patients with lumbar disc herniation, we recognize that our sample was limited by a cross-sectional design and convenience recruitment. Future studies should use random or stratified sampling to ensure the cohort more accurately represents the broader LDH population.

### 4.9. Clinical Implications

The substantial quality of life impairment caused by lumbar disc herniation has clear ramifications for primary care, rehabilitation, and health policy. The SF-36 proved effective at distinguishing high-risk groups (α = 0.922). Older adults, women, and patients reporting severe pain exhibited considerably elevated odds of severe daily activity limitation, with intense pain conferring more than fourfold increased risk. These findings support the integration of SF-36 screening into routine primary care to facilitate early identification, triage, and referral for at-risk patients. Published data [28] evidenced SF-36’s prognostic value in musculoskeletal conditions, suggesting that the SF-36 could strengthen community-based risk stratification and resource allocation. In our sample, 26.7% experienced severe daily activity limitations, and 62.5% scored poorly on the physical/emotional composite—highlighting a critical need for targeted rehabilitation resources. HRQoL findings could help prioritize multidisciplinary program funding in regions and demographic groups bearing the greatest burden [24]. The most affected SF-36 domains, bodily pain, physical functioning, and vitality, can inform the content of rehabilitation strategies, emphasizing intensive pain management, functional restoration, and psychosocial support. The results support biopsychosocial care models informed by HRQoL assessment, consistent with Romanian analyses calling for patient-reported outcomes to guide clinical decisions [29]. Evidence from Liu et al. (2022) [25] and Noormohammadpour et al. (2018) [26] supports the efficacy of interventions like core stability training and therapeutic climbing in improving SF-36 scores among chronic low back pain patients. In addition to functional improvements, Yu et al. (2023) [30] reported that quality of life increases with Pilates, indicating that HRQoL monitoring might enhance rehabilitation procedures and slow the development of impairment.

### 4.10. Limitations

This study has several limitations. First, the cross-sectional design precludes causal inference. Second, recruitment was conducted through clinical networks and patient support groups, yielding a community-based convenience sample rather than a randomly selected population cohort, which may limit generalizability. Third, a disease-specific disability instrument such as the Oswestry Disability Index (ODI) was not applied, so complementary insights into spinal-specific functional impairment are lacking. Fourth, our model did not include comorbidities, socioeconomic factors, or herniation severity, which likely limited its explanatory power and indicates that additional unmeasured variables contribute to functional limitation. Fifth, performance measures such as Nagelkerke R^2^ or classification accuracy were not reported for the logistic regression model; therefore, although associated factors were identified, the overall explanatory power of the model could not be fully assessed. Sixth, assumptions of regression models, including residual normality, homoscedasticity, and multicollinearity, were not formally tested, which may limit the robustness of model interpretation. Seventh, no adjustment for multiple comparisons was applied, given the exploratory nature of domain- and item-level analyses; thus, marginal associations should be interpreted with caution, although the key findings (pain, age, and sex effects) were highly significant and robust. Eighth, the sample size was calculated using the general population of Iași County as the denominator, which may not reflect the true population of LDH patients. The chosen 9% margin of error is relatively high and should be viewed as a feasibility compromise rather than a precise epidemiological estimate. Ninth, most participants were older adults, and the study did not specify whether cases were predominantly chronic, acute, pre- or post-surgical, which further limits the generalizability of findings to the broader LDH population. Finally, all predictors were entered into the regression models as main effects without interaction terms, given the limited sample size. A key limitation of this study concerns the use of the Physical and Emotional Status (PES) score, which was developed as an exploratory composite derived from selected vitality and mental health items of the SF-36. The PES is not a standardized SF-36 subscale, and therefore its findings should be interpreted descriptively, rather than as psychometrically confirmed outcomes. Although it provided a useful summary of the combined physical vitality and emotional well-being of lumbar disc herniation patients, its construct validity, reliability, and external applicability should be formally assessed in larger and more diverse samples in future research.

Together, these limitations highlight that daily activity limitation is influenced by multiple factors beyond those captured in our analysis.

## 5. Conclusions

LDH substantially impairs quality of life across physical, emotional, and social domains. Pain and functional restrictions are major contributors to this decline, but other significant factors include decreased energy, social disengagement, and negative health attitudes. Our findings demonstrate strong SF-36 reliability (α = 0.922) and suggest the DAL composite could identify patients vulnerable to severe functional and psychosocial deterioration—supporting its integration into community screening, rehabilitation strategies, and health policy in Romania. The following studies should use a biopsychosocial framework that integrates verified psychological, social, and cultural indices with clinical data. This approach would help develop more individualized and interdisciplinary care plans and allow for a more thorough understanding of the causes behind HRQoL deterioration in individuals with LDH.

## Figures and Tables

**Table 1:** DAL in relation to demographic and health characteristics.

Characteristic	Severe Limitations (*n* = 32)	Moderate Limitation (*n* = 58)	Mild Limitation (*n* = 30)	Chi-Square Test Likelihood Ratio (*p*)
**Female sex**	18 (56.3%)	32 (55.2%)	14 (46.7%)	*p* = 0.697
**Age > 60 years**	18 (56.3%)	40 (69.0%)	12 (40.0%)	*p* = 0.031
**Self-rated health**				*p* = 0.001
Excellent	0 (0.0%)	5 (8.6%)	3 (10.0%)	
Very good	5 (15.6%)	10 (17.2%)	9 (30.0%)	
Good	6 (18.8%)	19 (32.8%)	17 (56.7%)	
Fair	13 (40.6%)	19 (32.8%)	1 (3.3%)	
Poor	8 (25.0%)	5 (8.6%)	0 (0.0%)	
**Health compared to one year ago**				*p* = 0.002
Much better	5 (15.6%)	20 (34.5%)	9 (30.0%)	
Better	4 (12.5%)	12 (20.7%)	8 (26.7%)	
About the same	5 (15.6%)	13 (22.4%)	11 (36.7%)	
Somewhat worse	12 (37.5%)	8 (13.8%)	2 (6.7%)	
Much worse	6 (18.8%)	8 (8.6%)	0 (0.0%)	

**Table 2:** Multivariate linear regression. Dependent variable: DAL Score. Independent variables: responses to the questions on age, general health status, and current health compared to one year ago.

Model	R	R Square	Adjusted R Square	Std. Error of the Estimate	R Square Change	F Change	df1	df2	Sig. F Change
1	0.101 (a)	0.010	0.002	0.721	0.010	10.222	1	118	0.271
2	0.409 (b)	0.167	0.153	0.664	0.157	220.049	1	117	0.001
3	0.432 (c)	0.187	0.166	0.659	0.020	20.784	1	116	0.001

(a) Predictors: (Constant), Age. (b) Predictors: (Constant), Age, Your general health. (c) Predictors: (Constant), Age, Your general health, Your current health compared to one year ago.

**Table 3:** Regression coefficients for Model 3 predicting the DAL score.

Model 3	Unstandardized Coefficients		t	Sig.	95% Confidence Interval for B	
	B	Std. Error			Lower Bound (−95%CI)	Upper Bound (+95%CI)
(Constant)	2.98	0.294	10.151	0.001	2.398	3.561
Age	–0.001	0.004	–0.256	0.799	–0.009	0.007
General health	–0.224	0.063	–3.564	0.001	–0.349	–0.100
Current health	–0.086	0.051	–3.668	0.001	–0.387	–0.016

Dependent variable: DAL Score.

**Table 4:** Correlation between DAL and work-related problems in the past four weeks due to health status.

Problems in the Past 4 Weeks	Severe Limitation (*n* = 32)	Moderate Limitation (*n* = 58)	Mild Limitation (*n* = 30)	Chi-Square Test Likelihood Ratio
Reduced working time	32 (100%)	47 (81.0%)	9 (30.0%)	*p* = 0.001
Fewer activities	32 (100%)	47 (81.0%)	13 (43.3%)	*p* = 0.001
Limitations in type of work	30 (93.8%)	41 (70.7%)	8 (26.7%)	*p* = 0.001
Difficulties at work	32 (100%)	48 (82.8%)	9 (30.0%)	*p* = 0.001

**Table 5:** Correlation between DAL and problems experienced in the past four weeks due to emotional issues.

Problems in the Past 4 Weeks	Severe Limitation (*n* = 32)	Moderate Limitation (*n* = 58)	Mild Limitation (*n* = 30)	Chi-Square Test Likelihood Ratio
Reduced working time	29 (90.6%)	45 (77.6%)	12 (40.0%)	*p* = 0.001
Fewer activities	29 (90.6%)	47 (81.0%)	11 (36.7%)	*p* = 0.001
Difficulty maintaining the same level of attention or care	30 (93.8%)	46 (79.3%)	12 (40.0%)	*p* = 0.001

**Table 6:** Correlation between DAL and the impact on social activities in the past four weeks due to physical or emotional health problems.

Impact on Social Activities in the Past 4 Weeks	Severe Limitation (*n* = 32)	Moderate Limitation (*n* = 58)	Mild Limitation (*n* = 30)	Chi-Square Test Likelihood Ratio
Very much	14 (43.8%)	9 (15.5%)	1 (3.3%)	*p* = 0.001
A lot	11 (34.4%)	23 (39.7%)	2 (6.7%)	
Moderate	6 (18.8%)	19 (32.8%)	7 (23.3%)	
A little	1 (3.1%)	5 (8.6%)	13 (43.3%)	
Not at all	0 (0.0%)	2 (3.4%)	7 (23.3%)	

**Table 7:** Correlation between DAL and bodily pain during the past four weeks.

Bodily Pain in the Past 4 Weeks	Severe Limitation (*n* = 32)	Moderate Limitation (*n* = 58)	Mild Limitation (*n* = 30)	Chi Square Test Likelihood Ratio
None	0 (0.0%)	0 (0.0%)	3 (10.0%)	*p* = 0.001
Very mild	0 (0.0%)	1 (1.7%)	11 (36.7%)	
Mild	0 (0.0%)	5 (8.6%)	5 (8.6%)	
Moderate	5 (15.6%)	25 (43.1%)	9 (30.0%)	
Severe	18 (56.3%)	20 (34.5%)	2 (6.7%)	
Very severe	9 (28.1%)	7 (12.1%)	0 (0.0%)	

**Table 8:** Correlation between DAL and the impact of physical or emotional health problems on usual work in the past four weeks.

Impact on Usual Work in the Past 4 Weeks	Severe Limitation (*n* = 32)	Moderate Limitation (*n* = 58)	Mild Limitation (*n* = 30)	Chi Square Test Likelihood Ratio *p*
Very much	11 (34.4%)	8 (13.8%)	0 (0.0%)	*p* = 0.001
A lot	17 (53.1%)	25 (43.1%)	2 (6.7%)	
Moderate	3 (9.4%)	21 (36.2%)	9 (30.0%)	
A little	1 (3.1%)	4 (6.9%)	13 (43.3%)	
Not at all	0 (0.0%)	0 (0.0%)	6 (20.0%)	

**Table 9:** Correlation between physical and emotional status and demographic characteristics and health status of the study group.

Characteristics	Lower Score—Better Status (*n* = 45)	Higher Score—Worse Status (*n* = 75)	Chi^2^ Test
**Female sex**	25 (55.6%)	39 (52.0%)	*p* = 0.426
**Age > 60 years**	25 (55.6%)	45 (60.0%)	*p* = 0.386
**Self-rated health**			*p* = 0.357
Excellent	2 (4.4%)	6 (8.0%)	
Very good	10 (22.2%)	14 (18.7%)	
Good	14 (31.1%)	28 (37.3%)	
Fair	11 (24.4%)	22 (29.3%)	
Poor	8 (17.8%)	5 (6.7%)	
**Current health compared to one year ago**			*p* = 0.046
Much better	10 (22.2%)	20 (26.7%)	
Better	12 (26.7%)	12 (16.0%)	
About the same	9 (20.0%)	24 (32.0%)	
Somewhat worse	6 (13.3%)	16 (21.3%)	
Much worse	8 (17.8%)	3 (4.0%)	
**Limitation of daily activities**			*p* = 0.133
Severe	15 (33.3%)	17 (22.7%)	
Moderate	23 (51.1%)	35 (46.7%)	
Mild	7 (15.6%)	23 (30.7%)	
**Impact on social activities**			*p* = 0.05
Very much	12 (26.7%)	12 (16.0%)	
A lot	14 (31.1%)	22 (29.3%)	
Moderate	13 (28.9%)	19 (25.3%)	
A little	4 (8.9%)	15 (20.0%)	
Not at all	2 (4.4%)	7 (9.3%)	
Bodily pain			*p* = 0.009
None	0 (0.0%)	3 (4.0%)	
Very mild	3 (6.7%)	9 (12.0%)	
Mild	3 (6.7%)	7 (9.3%)	
Moderate	12 (26.7%)	27 (36.0%)	
Severe	17 (37.8%)	23 (30.7%)	
Very severe	10 (22.2%)	6 (8.0%)	
**Impact on usual work**			*p* = 0.212
Not at all	1 (2.2%)	5 (6.7%)	
A little	7 (15.6%)	11 (17.7%)	
Moderate	8 (17.8%)	25 (33.3%)	
A lot	20 (44.4%)	24 (32.0%)	
Very much	9 (20.0%)	10 (13.3%)	

**Table 10:** Multivariate linear regression. Dependent variable: PES score. Independent variables: responses to questions on sex, age, general health, current health compared to one year ago, daily activity limitation, impact on social activities, bodily pain, and impact on usual work.

Model	R	R Square	Adjusted R Square	Std. Error of the Estimate	Statistical Test				
					R Square Change	F Change	df1	df2	Sig. F Change
1	0.046 (a)	0.002	0.006	3.277	0.002	0.249	1	118	0.619
2	0.087 (b)	0.008	0.009	3.282	0.006	0.649	1	117	0.422
3	0.099 (c)	0.010	0.016	3.292	0.002	0.246	1	116	0.621
4	0.106 (d)	0.011	0.023	3.304	0.002	0.182	1	115	0.670
5	0.129 (e)	0.017	0.026	3.309	0.005	0.624	1	114	0.431
6	0.150 (f)	0.022	0.029	3.314	0.006	0.669	1	113	0.415
7	0.203 (g)	0.041	0.019	3.297	0.019	2.209	1	112	0.140
8	0.244 (h)	0.060	0.008	3.279	0.018	2.173	1	111	0.143

Notes: (a) Predictors: (Constant), Sex. (b) Predictors: (Constant), Sex, Age. (c) Predictors: (Constant), Sex, Age, General health. (d) Predictors: (Constant), Sex, Age, General health, Current health compared to one year ago. (e) Predictors: (Constant), Sex, Age, General health, Current health, DAL score. (f) Predictors: (Constant), Sex, Age, General health, Current health, Daily activity limitation, Social activities. (g) Predictors: (Constant), Sex, Age, General health, Current health, Daily activity limitation, Social activities, Bodily pain. (h) Predictors: (Constant), Sex, Age, General health, Current health, Daily activity limitation, Social activities, Bodily pain, Impact on usual work.

**Table 11:** Correlation between the response to the statement ‘My health is excellent’ and demographic characteristics and health status.

Characteristics	False (*n* = 57)	True/Don’t Know (*n* = 63)	Chi^2^ Test (*p*)
**Female sex**	36 (63.2%)	28 (44.4%)	*p* = 0.031
**Age > 60 years**	42 (73.7%)	28 (44.4%)	*p* = 0.001
**Self-rated health**			*p* = 0.001
Excellent	1 (1.8%)	7 (11.1%)	
Very good	7 (12.3%)	17 (27.0%)	
Good	10 (17.5%)	32 (50.8%)	
Fair	26 (45.6%)	7 (11.1%)	
Poor	13 (22.8%)	0 (0.0%)	
**Health compared to one year ago**			*p* = 0.01
Much better	11 (19.3%)	23 (36.5%)	
Better	11 (19.3%)	13 (20.6%)	
About the same	13 (22.8%)	16 (25.4%)	
Somewhat worse	12 (21.2%)	10 (15.9%)	
Much worse	10 (17.5%)	1 (1.6%)	
**Limitations in daily activities**			*p* = 0.001
Severe	24 (42.1%)	8 (12.7%)	
Moderate	49 (49.1%)	30 (47.6%)	
Mild	5 (8.8%)	25 (39.7%)	
**Impact on social activities**			*p* = 0.001
None	0 (0.0%)	9 (14.3%)	
Slight	3 (5.3%)	16 (25.4%)	
Moderate	23 (40.4%)	9 (14.3%)	
Severe	16 (28.1%)	20 (31.7%)	
Very severe	15 (26.3%)	9 (14.3%)	

**Table 12:** Correlation between the response to the statement ‘I expect my health to deteriorate’ and demographic characteristics and health status.

Characteristics	True (*n* = 39)	False/Don’t Know (*n* = 81)	Chi^2^ Test (*p*)
**Female sex**	25 (64.1%)	39 (48.1%)	*p* = 0.074
**Age > 60 years**	27 (69.2%)	43 (53.1%)	*p* = 0.068
**Self-rated health**			*p* = 0.001
Excellent	1 (2.6%)	7 (8.6%)	
Very good	5 (12.8%)	19 (23.5%)	
Good	4 (10.3%)	38 (46.9%)	
Fair	19 (48.7%)	14 (17.3%)	
Poor	10 (25.6%)	3 (3.7%)	
**Health compared to one year ago**			*p* =0.001
Much better	4 (10.3%)	30 (37.0%)	
Better	5 (12.8%)	19 (23.5%)	
About the same	12 (30.8%)	17 (21.0%)	
Somewhat worse	10 (25.6%)	12 (14.8%)	
Much worse	8 (20.5%)	3 (3.7%)	
**Limitations in daily activities**			*p* = 0.001
Severe	18 (46.2%)	14 (7.3%)	
Moderate	19 (48.7%)	39 (48.1%)	
Mild	2 (5.1%)	28 (34.6%)	
**Impact on social activities**			*p* = 0.012
None	2 (5.1%)	7 (8.6%)	
Slight	1 (2.6%)	18 (22.2%)	
Moderate	10 (25.6%)	22 (27.2%)	
Severe	14 (35.9%)	22 (27.2%)	
Very severe	12 (30.8%)	12 (14.8%)	

**Table 13:** Correlation between the response to the statement ‘I am as healthy as anyone I know’ and demographic characteristics and health status.

Characteristics	False (*n* = 46)	True/Don’t Know (*n* = 74)	Chi^2^ Test (*p*)
**Female sex**	27 (58.7%)	37 (50.0%)	*p* = 0.230
**Age > 60 years**	34 (73.9%)	36 (48.6%)	*p* = 0.005
**Self-rated health**			*p* = 0.001
Excellent	1 (2.2%)	7 (9.5%)	
Very good	7 (15.2%)	17 (23.0%)	
Good	8 (17.4%)	34 (45.9%)	
Fair	21 (45.7%)	12 (16.2%)	
Poor	9 (19.6%)	4 (5.4%)	
**Health compared to one year ago**			*p* = 0.028
Much better	9 (19.6%)	25 (33.8%)	
Better	10 (21.7%)	14 (18.9%)	
About the same	10 (21.7%)	19 (25.7%)	
Somewhat worse	9 (19.6%)	13 (17.6%)	
Much worse	8 (17.4%)	3 (4.1%)	
**Limitations in daily activities**			*p* = 0.001
Severe	19 (41.3%)	13 (17.6%)	
Moderate	23 (50.0%)	35 (47.3%)	
Mild	4 (8.7%)	26 (35.1%)	
**Impact on social activities**			*p* = 0.001
None	0 (0.0%)	9 (12.2%)	
Slight	3 (6.5%)	16 (21.6%)	
Moderate	14 (30.4%)	18 (24.3%)	
Severe	15 (32.6%)	21 (28.4%)	
Very severe	14 (30.4%)	10 (13.5%)	

**Table 14:** Correlation between the response to the statement ‘I get sick more easily than other people’s and demographic characteristics and health status.

Characteristics	True (*n* = 42)	False/Don’t Know (*n* = 78)	Chi^2^ Test (*p*)
**Female sex**	25 (59.5%)	39 (50.0%)	*p* = 0.210
**Age > 60 years**	28 (66.7%)	42 (53.8%)	*p* = 0.122
**Self-rated health**			*p* = 0.001
Excellent	1 (2.4%)	7 (9.0%)	
Very good	5 (11.6%)	19 (24.4%)	
Good	11 (26.2%)	31 (39.7%)	
Fair	14 (33.3%)	19 (24.4%)	
Poor	11 (26.2%)	2 (2.6%)	
**Health compared to one year ago**			*p* = 0.001
Much better	8 (19.0%)	26 (33.3%)	
Better	9 (21.4%)	15 (16.2%)	
About the same	6 (14.3%)	23 (29.5%)	
Somewhat worse	8 (19.0%)	14 (17.9%)	
Much worse	11 (26.2%)	0 (0.0%)	
**Limitations in daily activities**			*p* = 0.001
Severe	20 (47.6%)	12 (15.4%)	
Moderate	18 (42.9%)	40 (51.3%)	
Mild	4 (9.5%)	26 (33.3%)	
**Impact on social activities**			*p* = 0.001
None	0 (0.0%)	9 (11.5%)	
Slight	1 (2.4%)	18 (23.1%)	
Moderate	11 (26.2%)	21 (26.9%)	
Severe	16 (38.1%)	20 (25.6%)	
Very severe	14 (33.3%)	10 (12.8%)	

## Data Availability

All data from the first author and the corresponding author are available.

## Supplementary Materials

The following supporting information can be downloaded at: https://www.mdpi.com/article/10.3390/jcm14228258/s1, Table S1: Summary of SF-36 domains by gender and age group (Q1–Q8).

## Abbreviations

The following abbreviations are used in this manuscript:

%	Percentage
ANOVA	Analysis of Variance
AUC	Area Under the Curve
B	Unstandardized Regression Coefficient
CI	Confidence Interval
COVID-19	Coronavirus Disease 2019
DAL	Daily Activity Limitation
df1	Degrees of Freedom (numerator, regression predictor)
df2	Degrees of Freedom (denominator, residual error)
GH	General Health
HCWs	Healthcare Workers
HRQoL	Health-Related Quality of Life
LDH	Lumbar Disc Herniation
n	Number of Participants/Respondents
ODI	Oswestry Disability Index
OR	Odds Ratio
PES	Physical and Emotional Status
QOL	Quality of Life
ROC	Receiver Operating Characteristic
SD	Standard Deviation
SF-36	36-Item Short Form Health Survey
Sig.	Significance (p-value)
Sig. F Change	Significance of the F Change Test (p-value for added predictors in regression)
SPSS	Statistical Package for the Social Sciences
SRH	Self-Rated Health
Std. Error	Standard Error of the Coefficient
*t*	*t*-statistic (Student’s *t*-test value for regression coefficient)
UTI	Urinary Tract Infection
VAS	Visual Analogue Scale
