# Supplementary material for: Associations Between Health-Related Quality of Life and Clinical Factors in Lumbar Disc Herniation: Evidence from a Romanian Cohort Using the SF-36
Source: J Clin Med. 2025 Nov 20;14(22):8258. doi: 10.3390/jcm14228258 (PMC12653116; doi:10.3390/jcm14228258)
Supplement: Supplementary file 1 [file jcm-14-08258-s001.zip › jcm-3936108-supplementary.pdf]

Supplementary Table S1. Summary of SF-36 domains by gender and age group (Q1–Q8).

**Self-Rated Health**	**Men (%)**	**Women (%)**	**≤60 y.o. (%)**	**>60 y.o. (%)**
Excellent	5.03	1.68	2.51	4.19
Very Good	12.50	7.50	9.16	10.84
Good	17.50	17.50	19.99	15.02
Fair	7.51	19.98	8.33	19.17
Poor	4.16	6.64	1.66	9.14
**Perceived change in self-rated health over the past year.**	**Men (%)**	**Women (%)**	**≤60 y.o. (%)**	**>60 y.o. (%)**
Much better now	19.1308	9.1692	14.15	14.15
Somewhat better now	10.84	9.16	11.66	8.34
About the same	11.6886	12.5114	9.1718	15.0282
Somewhat worse now	4.1541	14.1459	4.9959	13.3041
Much worse now	0.8372	8.3628	1.6744	7.5256
**Work-related problems in the past four weeks due to physical health**	**Male (%)**	**Female (%)**	**≤ 60 years (%)**	**> 60 years (%)**
Reduced working time	47.7	52.3	38.6	61.4
Fewer activities performed	44.6	55.4	43.5	56.5
Limitations in the type of work	45.6	54.4	41.8	58.2
Difficulties in performing work	46.1	53.9	37.1	62.9
**Limitations of daily activities in the past four weeks due to emotional problems**	**Male (%)**	**Female (%)**	**≤ 60 years (%)**	**> 60 years (%)**
Reduced working time	33.34	38.36	26.67	45.03
Fewer activities performed	35.02	37.48	28.35	44.15
Difficulty paying attention and caring	33.42	39.95	27.49	45.81
**Impact of health or emotional problems on social activities in the past four weeks**	**Male**	**Female**	**≤ 60 years**	**> 60 years**

Very much	9.16	10.84	10.00	10.00
A lot	14.16	15.84	9.99	20.01
Moderate	12.52	14.18	7.50	19.20
A little	6.65	9.15	9.99	5.81
Not at all	4.17	3.33	4.17	3.33
**Bodily pain during the past four weeks**	**Male**	**Female**	**≤ 60 years**	**> 60 years**
None	1.67	0.83	1.67	0.83
Very mild	4.17	5.83	6.67	3.33
Mild	4.15	4.15	4.15	4.15
Moderate	14.17	18.33	8.32	24.18
Severe	14.15	19.15	13.32	19.98
Very severe	8.31	4.99	7.49	5.83
**Impact of bodily pain on usual work during the past four weeks**	**Male**	**Female**	**≤ 60 years**	**> 60 years**
Very much	8.31	7.49	7.49	8.31
A lot	16.70	20.00	16.70	20.00
Moderate	13.34	14.16	7.51	19.99
A little	5.00	10.01	6.66	8.34
Not at all	3.34	1.67	3.34	1.67
